# Supplementary material for: Single-cell transcriptomics reveal cellular diversity of aortic valve and the immunomodulation by PPARγ during hyperlipidemia
Source: Nat Commun. 2022 Sep 17;13:5461. doi: 10.1038/s41467-022-33202-2 (PMC9482653; doi:10.1038/s41467-022-33202-2)
Supplement: Supplementary file 1 — Supplementary Information [file 41467_2022_33202_MOESM1_ESM.pdf]

Supplementary Information

Single-cell transcriptomics reveal cellular diversity of aortic valve and the immunomodulation by PPARγ during hyperlipidemia

Seung Hyun Lee et al.

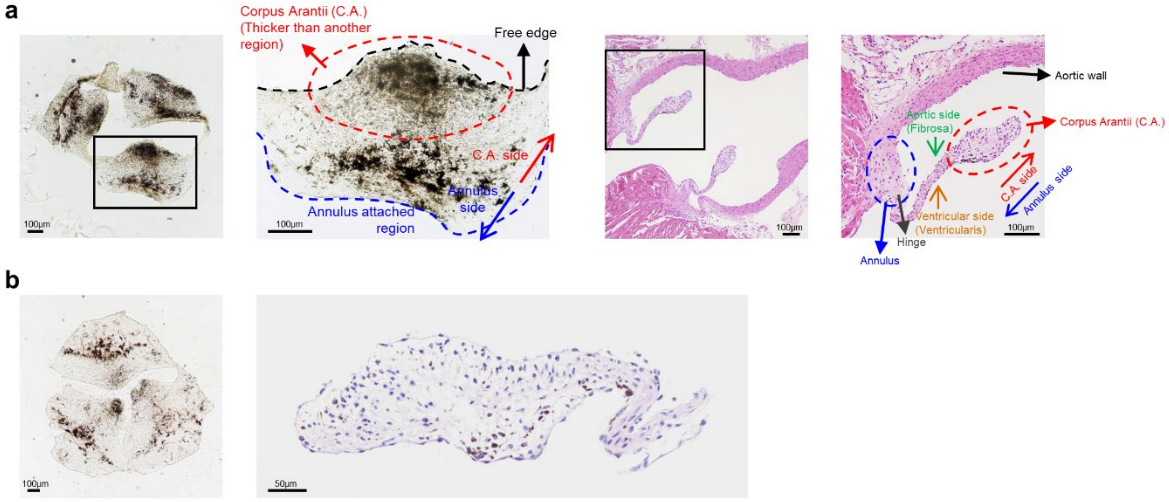

Supplementary Fig. 1. Histological description of mouse aortic valve.

a. Gross and histological features of mouse aortic valve. Whole-mount brightfield images (left), and H&E images (right). Black or dark brown colors are melanin pigments. Scale bar: 100μm. Data are representative of three independent experiments. **b.** Gross and histology of the aortic valve prepared for scRNA-seq and flow cytometric analysis. Aortic valves were carefully isolated from aortic sinus without contamination of other tissues. Whole-mount brightfield (left) and H&E images (right) of the isolated aortic valve. Black or dark brown spots are melanin pigments. Scale bar: 100 μm (left), 50 μm (right). Data are representative of three independent experiments.

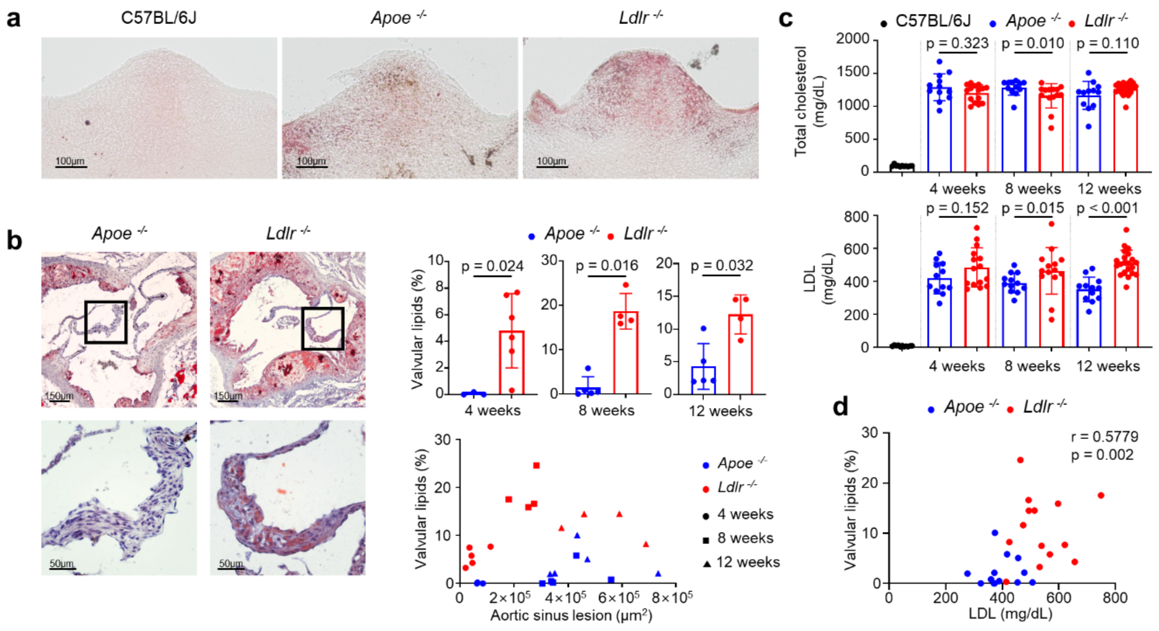

Supplementary Fig. 2. Comparison of aortic valvular lipid accumulation between WD-fed *Apoe*$^{-/-}$ and *Ldlr*$^{-/-}$ mice.

a, b. Lipid accumulation in the aortic valves of mice under normal conditions, C57BL/6J (wild type) fed normal chow diet or hyperlipidemic conditions, *Apoe*$^{-/-}$ and *Ldlr*$^{-/-}$ mice fed a western diet (WD). Whole-mount Oil Red O stain of the aortic valve of C57BL/6J (chow diet) or *Apoe*$^{-/-}$ and *Ldlr*$^{-/-}$ mice (WD for 16 weeks). Scale bar: 100 µm. **(a)** and comparison of aortic valvular lipid accumulation between *Apoe*$^{-/-}$ and *Ldlr*$^{-/-}$ mice (WD for 4, 8 or 12 weeks). Representative lipid stain images (left), measurement of valvular lipid deposition (right, top) and comparison with aortic sinus lesion (right, bottom) (*n* = 3 for *Apoe*$^{-/-}$, *n* = 6 for *Ldlr*$^{-/-}$ in 4 weeks; *n* = 5 for *Apoe*$^{-/-}$, *n* = 4 for *Ldlr*$^{-/-}$ in 8 and 12 weeks). Scale bar: 150 µm (left, top), 50 µm (left, bottom). **(b). c.** Total cholesterol and LDL levels in the blood plasma of C57BL/6J (chow diet) or *Apoe*$^{-/-}$, and *Ldlr*$^{-/-}$ mice (WD for 4, 8 or 12 weeks) (*n* = 11 for C57BL/6J; *n* = 12 for *Apoe*$^{-/-}$ in 4, 8, and 12 weeks; *n* = 15 for *Ldlr*$^{-/-}$ in 4 weeks, *n* = 14 for *Ldlr*$^{-/-}$ in 8 weeks, *n* =21 for *Ldlr*$^{-/-}$ in 12 weeks). **d.** Correlation between valvular lipid deposition and plasma LDL levels. Image data

are representative of three independent experiments unless otherwise stated. For **(b)** and **(c)**, two-sided Mann-Whitney test was used. For **(d)**, the Spearman correlation test was used. Data are presented as mean ± SD.

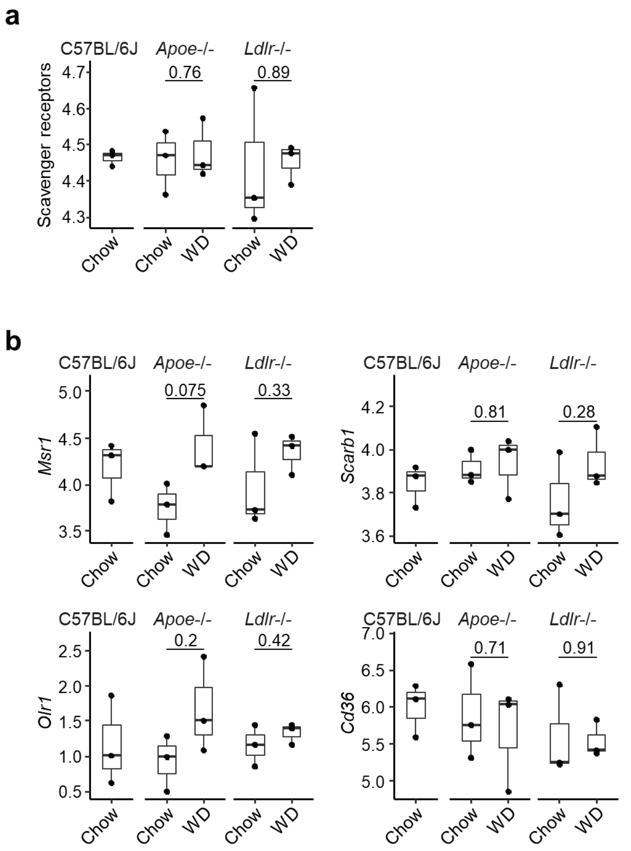

Supplementary Fig. 3. Expression levels of scavenger receptors in the aortic valve tissue of C57BL/6J, *Apoe*$^{-/-}$ and *Ldlr*$^{-/-}$ mice.

a. Boxplot of the average expression levels of scavenger receptor genes in the aortic valve tissue of C57BL/6J, *Apoe*$^{-/-}$, and *Ldlr*$^{-/-}$ mice (*Ager*, *Cd14*, *Cd163*, *Cd207*, *Cd36*, *Cd68*, *Clec7a*, *Colec12*, *Cxcl16*, *Ly75*, *Marco*, *Megf10*, *Mrc1*, *Msr1*, *Olr1*, *Scara3*, *Scara5*, *Scarb1*, *Scarb2*, *Scarf1*, *Scarf2*, *Ssc4d*, *Ssc5d*, *Stab1*, *Stab2*) (*n* = 3). **b.** Boxplots of the expression levels of each LDL/oxLDL scavenger receptor genes (*Msr1*, *Scarb1*, *Olr1*, and *Cd36*). Chow: chow diet-fed, WD: western diet-fed (for 8 weeks) (*n* = 3). Each box depicts the IQR and median of each score, whiskers indicate 1.5 times the IQR. p, two-sided T-test p-value.

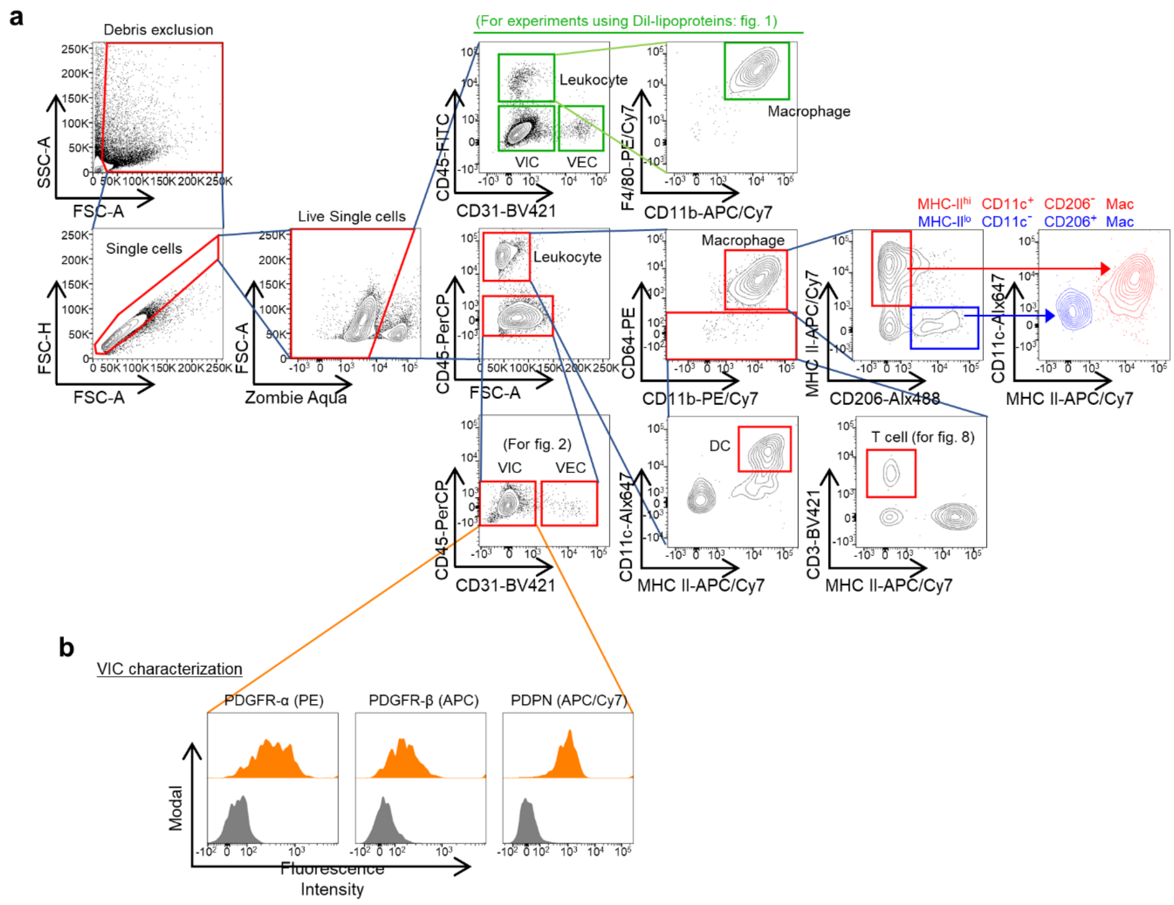

Supplementary Fig. 4. Gating strategy for flow cytometry analysis of aortic valvular cells.

a. Gating strategy of flow cytometry analysis of aortic valves. Alx488: Alexa Fluor 488. Alx647: Alexa Fluor 647. Mac: Macrophage. **b.** Characterization of VICs. Y-axis option of histograms: Modal (Normalized to mode).

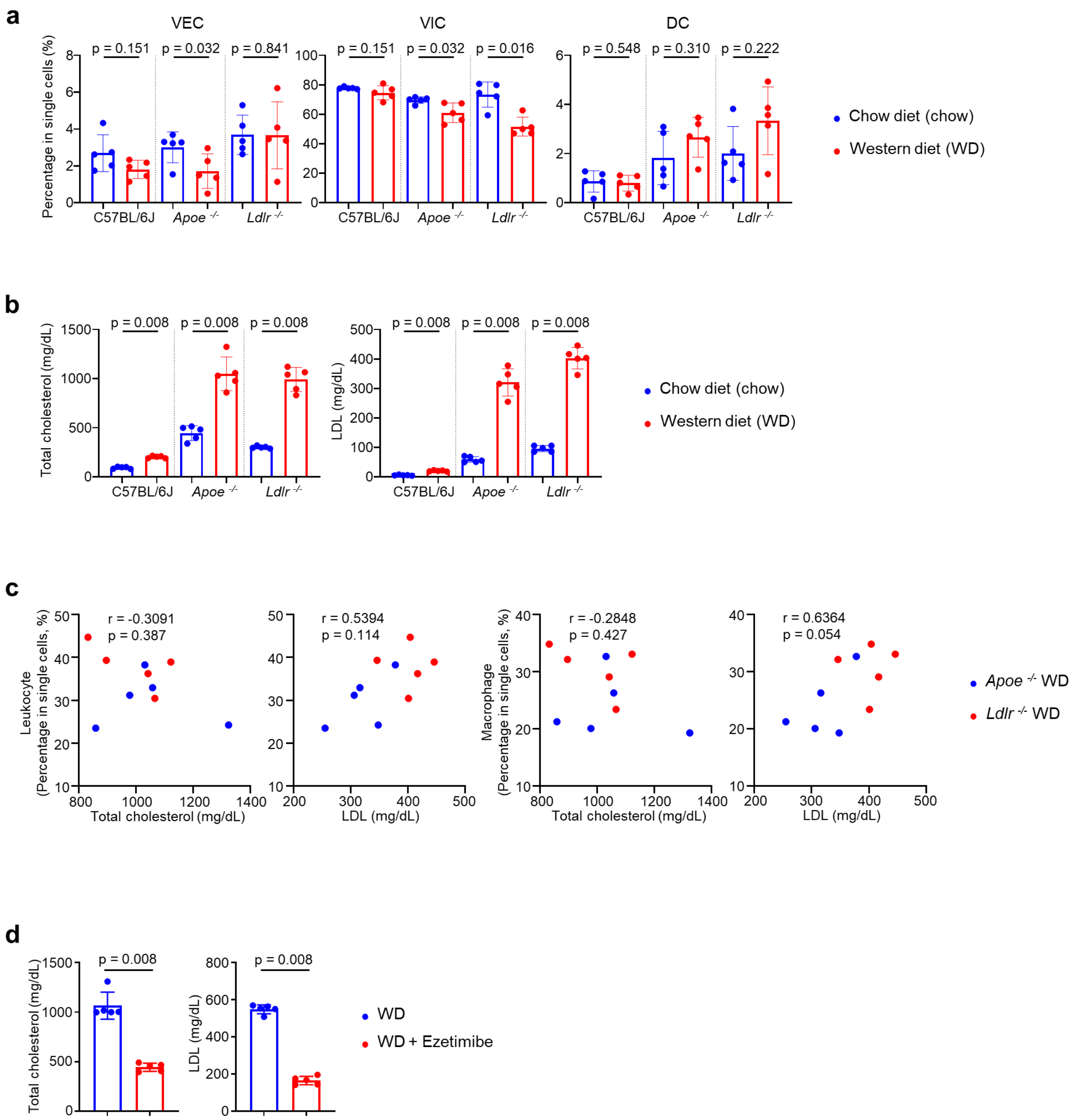

Supplementary Fig. 5. The relationship between lipid profiles and proportional changes in aortic valvular cells

a. Percentage of VECs, VICs, and DCs in the aortic valvular cells of chow-fed or WD-fed (for 10 weeks) C57BL/6J, *Apoe*$^{-/-}$, and *Ldlr*$^{-/-}$ mice (n = 5). **b.** Total cholesterol and LDL levels in the blood plasma of chow-fed or WD-fed (for 10 weeks) C57BL/6J, *Apoe*$^{-/-}$, and *Ldlr*$^{-/-}$ mice (n = 5). **c.** Correlations of total cholesterol and LDL levels with the percentage of leukocytes and

macrophages in the aortic valvular cells of WD-fed (for 10 weeks) hyperlipidemic mice (*Apoe*$^{-/-}$ and *Ldlr*$^{-/-}$). **d.** Total cholesterol and LDL levels in the blood plasma of normal WD-fed or ezetimibe containing WD-fed (for 10 weeks) *Ldlr*$^{-/-}$ mice (n = 5). This figure is extended data of Fig. 2. For **(a)**, **(b)**, and **(d)**, two-sided Mann-Whitney test was used. For **(c)**, the Spearman correlation test was used. Data are presented as mean ± SD.

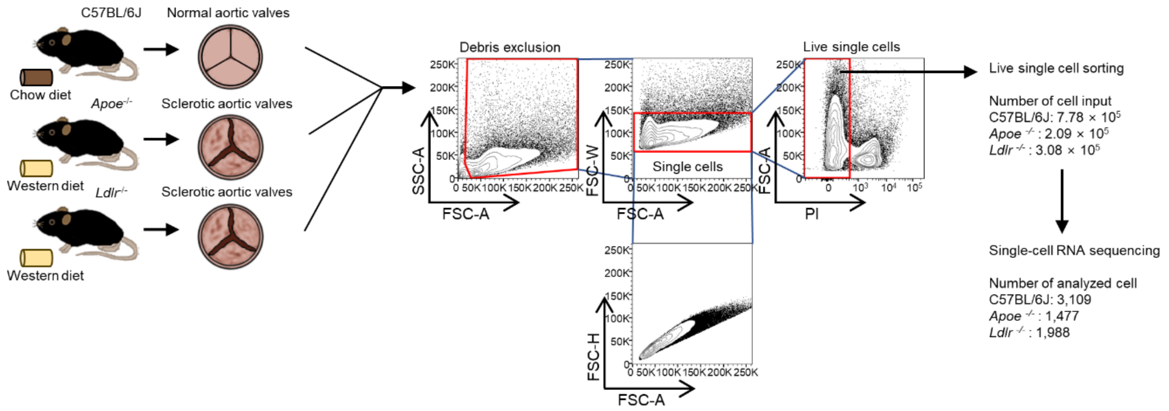

Supplementary Fig. 6. Cell sorting strategy for scRNA-seq analysis.

Experimental outline of the aortic valve scRNA sequencing with cell sorting strategy. Live single cells were sorted and used in scRNA-seq.

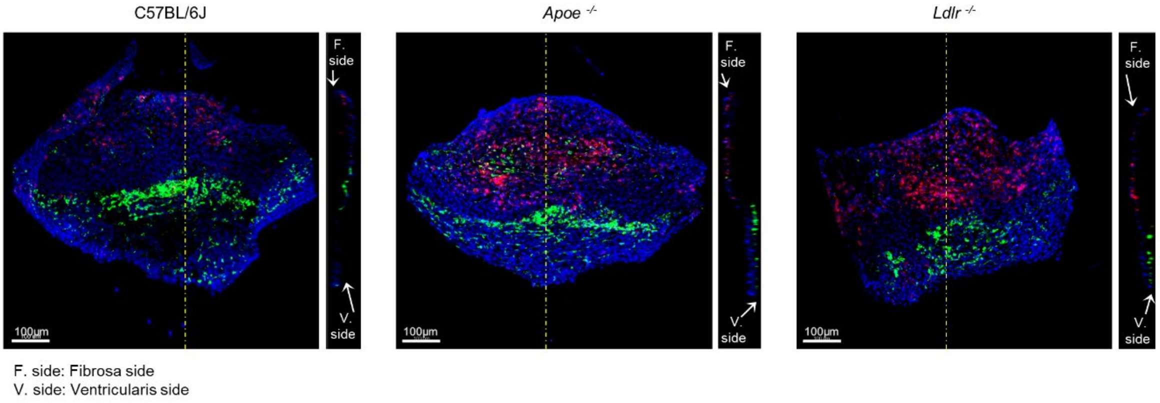

Supplementary Fig. 7. Spatial location of macrophage subsets.

Z-axis spatial localization of MHC-II^{hi} (red) macrophages and CD206^{+} (green) macrophages of aortic valve from C57BL/6J, *Apoe*$^{-/-}$ and *Ldlr*$^{-/-}$ mice. Original image (left), and lengthwise slice view (right). Scale bar: 100μm. Dashed line, plane of slice. This figure is extended data of Fig. 3e. Image data are representative of three independent experiments unless otherwise stated.

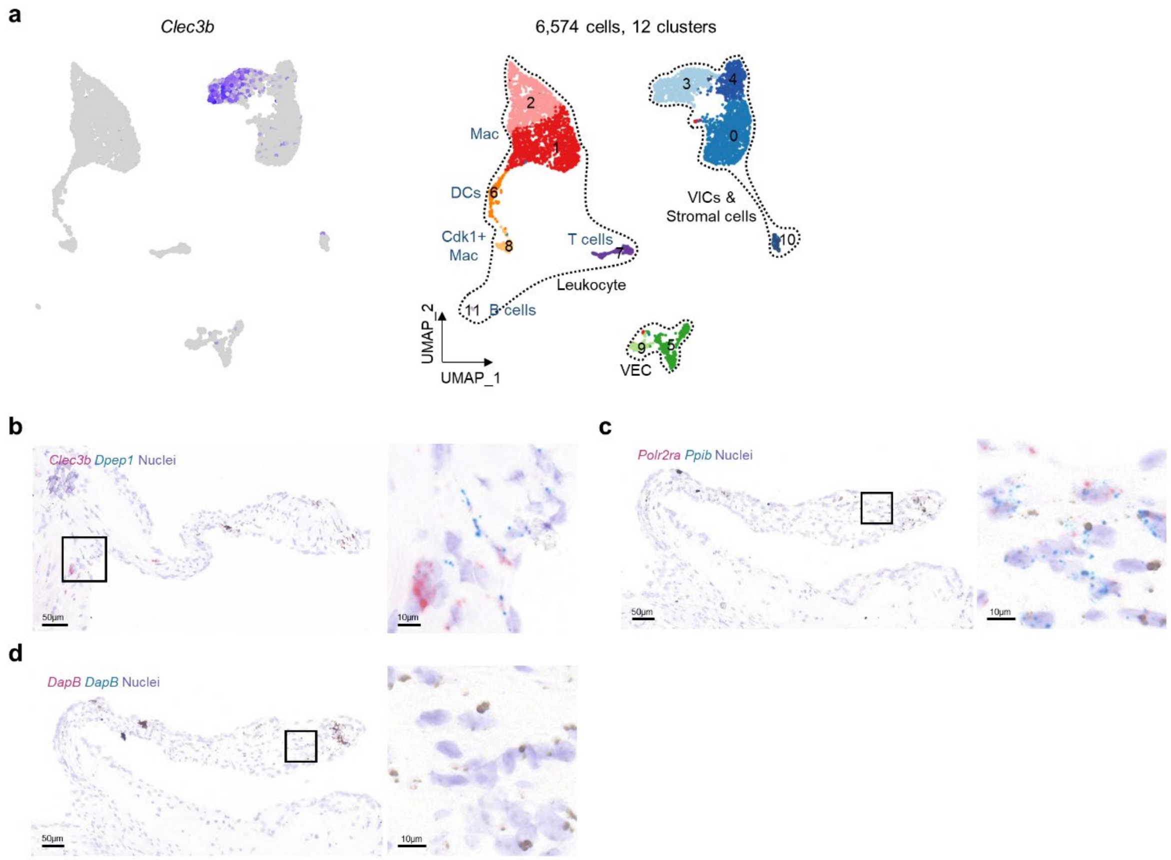

Supplementary Fig. 8. Location of *Clec3b*$^+$ *Dpep1*$^+$ fibroblasts.

a. UMAP plot of gene expression of *Clec3b* (gray to blue). Cluster_3 expressed a higher level of *Clec3b* than other clusters. **b-d.** RNA in situ hybridization of marker genes (*Clec3b*, *Dpep1*) of Cluster_3 (*Clec3b*$^+$ *Dpep1*$^+$ fibroblasts) with positive and negative control probes. Representative image of RNA in situ hybridization of *Clec3b* (red) and *Dpep1* (green) **(b)**, positive control probes: *Polr2ra* (red), *Ppib* (green) **(c)** and negative control probes: *DapB* (red and green) **(d)**. Scale bar: 50 µm (left), 10 µm (right). Cluster_3 cells (*Clec3b*$^+$ *Dpep1*$^+$ fibroblasts) were mainly located on the connective tissue adjacent to the hinge of aortic valve. Image data **(b-d)** are representative of at least three independent experiments.

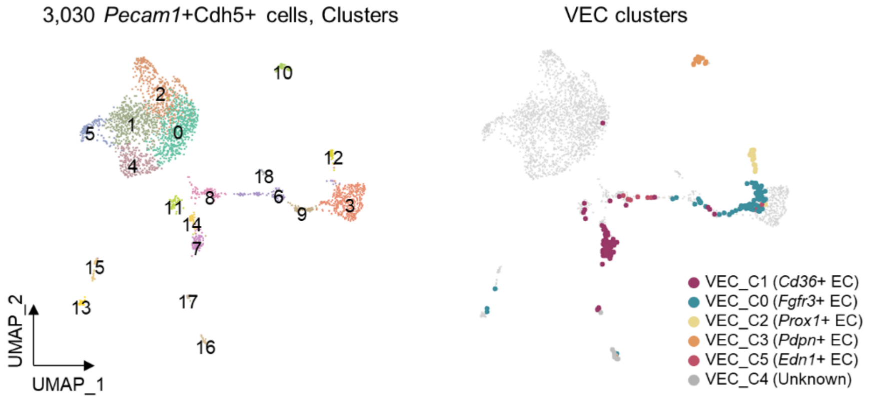

Supplementary Fig. 9. Meta-analysis of EC diversity from normal aortic ECs and VECs.

UMAP plot of 3,030 *Pecam1+Cdh5+* cells colored by the clusters (left) and VEC clusters (right). This figure is extended data of Fig. 6.

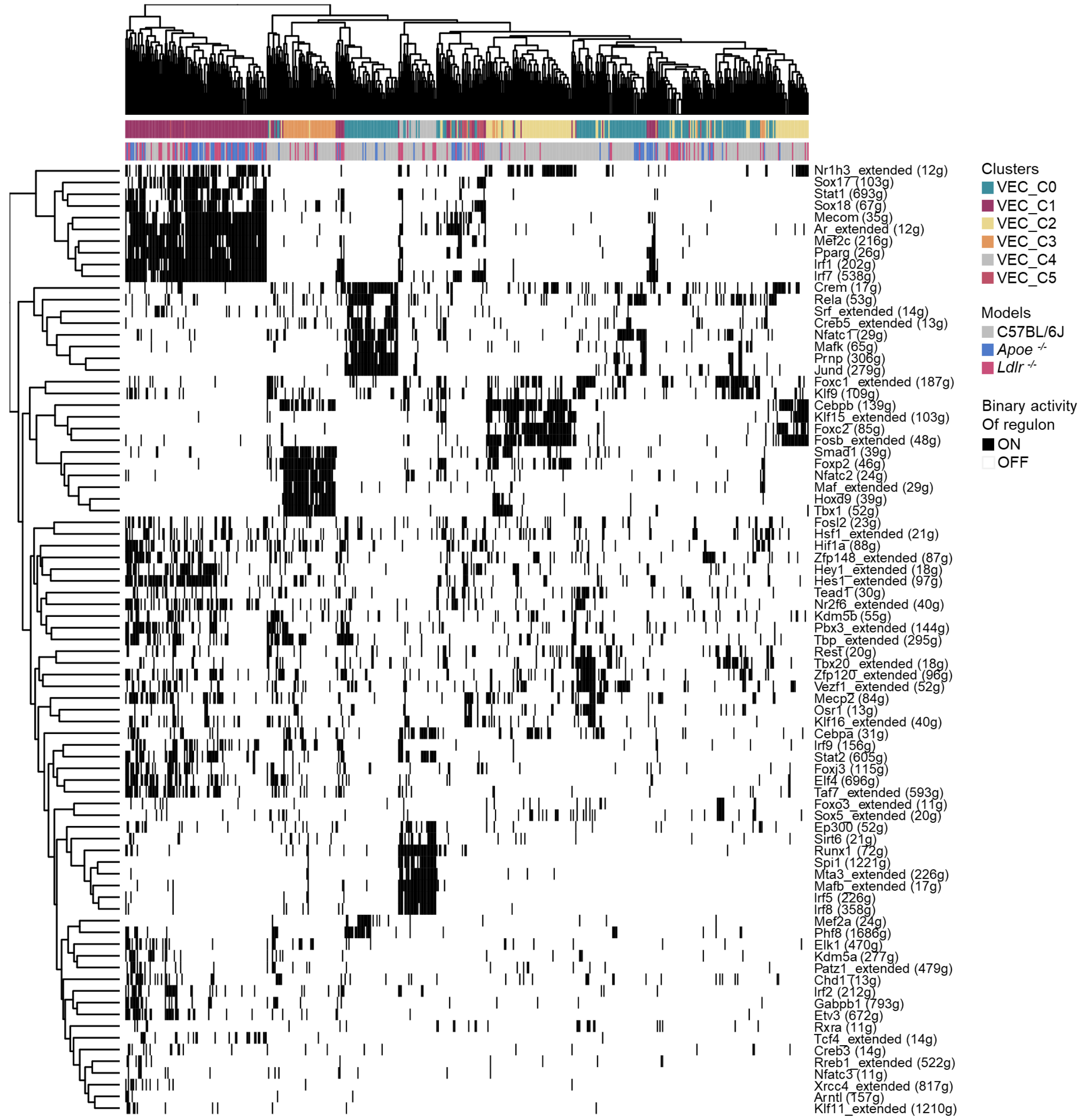

Supplementary Fig. 10. Heatmap of binary regulon activity for VECs.

Binary regulon activity is generated from the SCENIC AUC (area under curve) distribution for each cell in VECs.

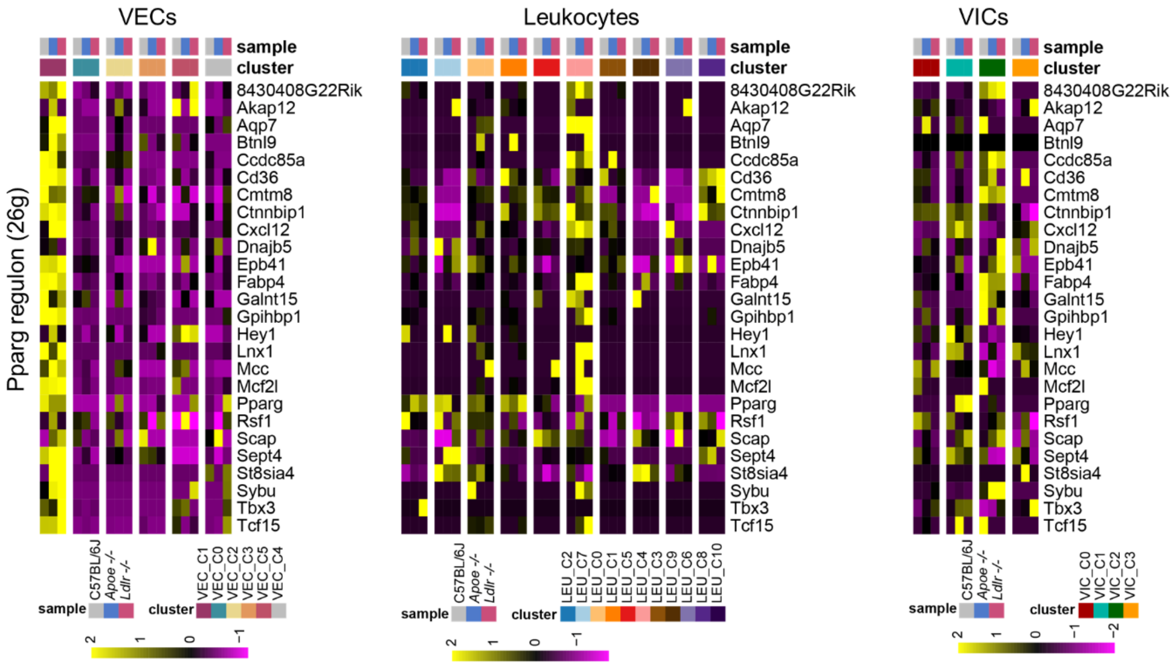

Supplementary Fig. 11. Heatmap of PPARγ regulon activities.

Expression map of genes in the PPARγ regulon, which is a gene module provided by SCENIC for each cluster of VECs, leukocytes, and VICs. Color represents average expression levels which are scaled by z-transformation and limited to a scale from -2 to 2. This figure is extended data of Fig. 7.

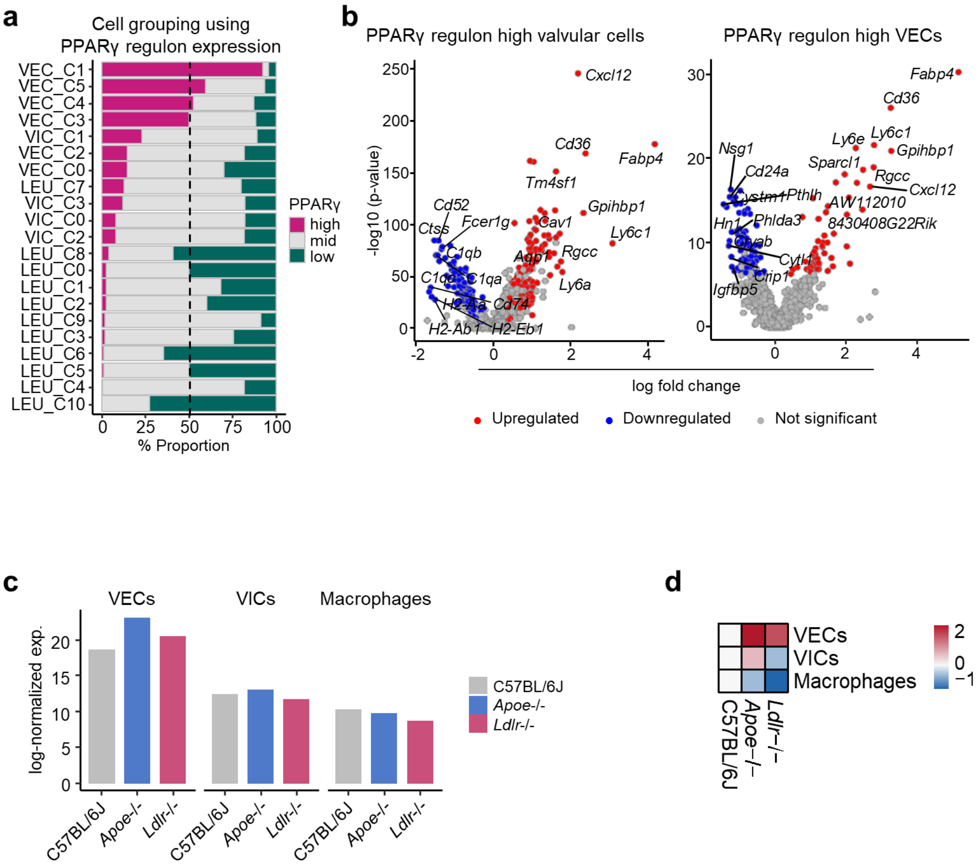

Supplementary Fig. 12. Analysis of PPARγ regulon and target genes.

a, b. Classification of aortic valvular cells based on average expression level of genes in PPARγ regulon. Percentage of cells grouped into PPARγ high, mid, and low in each cell cluster **(a)** and volcano plot comparing high versus low group in aortic valvular cells (left) and VECs (right) **(b)**. p-value, two-sided Wilcoxon rank sum test. **c.** Average expression levels of 49 PPARγ target genes (http://www.ppargene.org/) in VECs (VEC_C0, C1, and C2), VICs, and macrophages for each mouse model. **d.** Relative expression map of PPARγ target genes. Relative expression levels of PPARγ target genes in VECs (VEC_C0, C1, and C2), VICs, and macrophages of *Ldlr*$^{-/-}$ and *Apoe*$^{-/-}$ mice, compared with C57BL/6J mice, are shown as log fold changes.

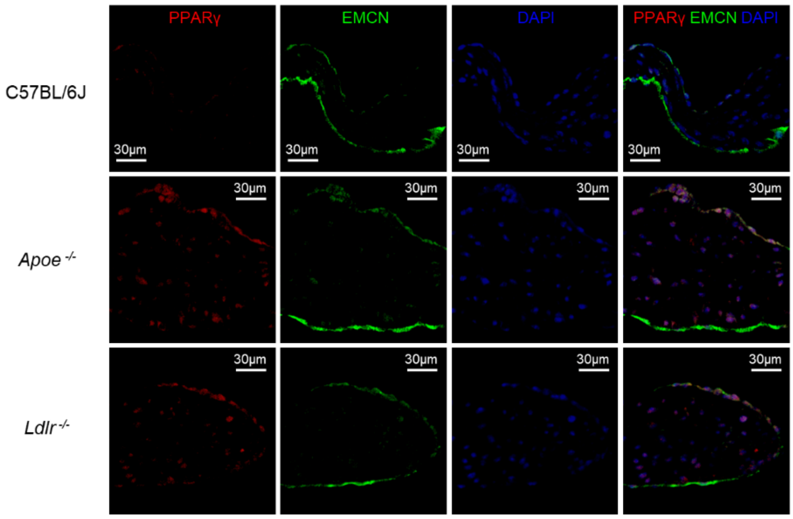

Supplementary Fig. 13. Immunostaining of PPARγ and EMCN in mouse aortic valve.

The color split series of each channel with merged image, shown in Fig. 7c. PPARγ (red), EMCN (green), and DAPI (blue). Scale bar: 30 μm. This figure is extended data of Fig. 7c. Each image dataset (*C57BL/6J*, *Apoe*$^{-/-}$, and *Ldlr*$^{-/-}$) is representative of four biologically independent samples.

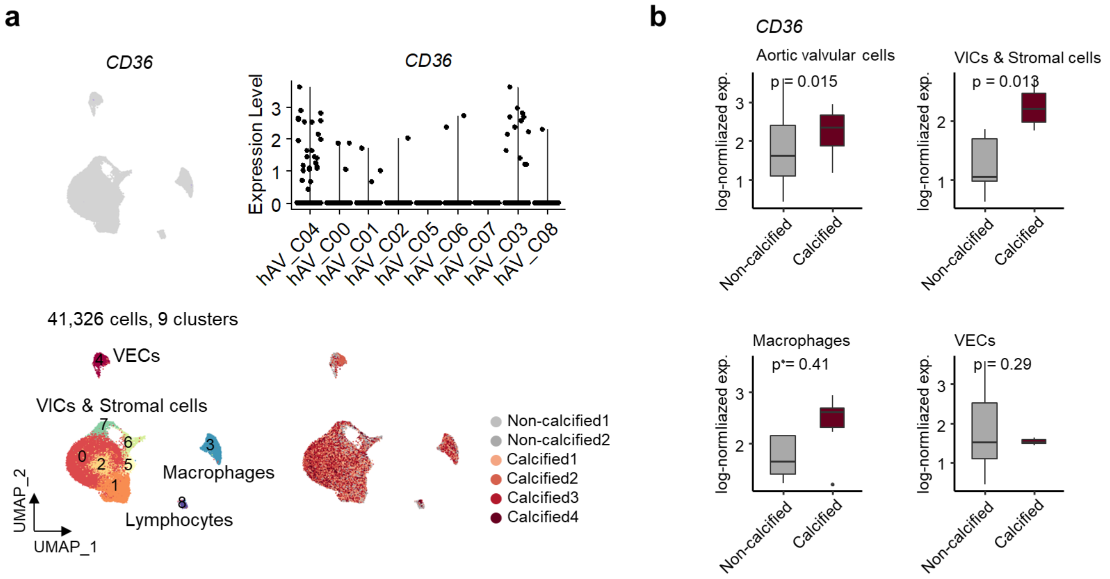

Supplementary Fig. 14. Expression level of *CD36* in scRNA-seq of human aortic valves.

a. Feature (left) and violin (right) plot of *CD36* expression in each cluster of single-cells derived from human aortic valve. **b.** Comparison of *CD36* expression in calcified versus non-calcified groups for aortic valvular cells (top, left), VICs and stromal cells (top, right), macrophages (bottom, left), and VECs (bottom, right). Cells having no expression for *CD36* were excluded (n=14 and 35 cells for aortic valvular cells; 4 and 5 cells for VICs and stromal cells; 8 and 5 cells for macrophages; 2 and 24 cells for VECs in calcified and non-calcified groups). Each box depicts the IQR and median of each score, whiskers indicate 1.5 times the IQR. p, two-sided T-test p-value.

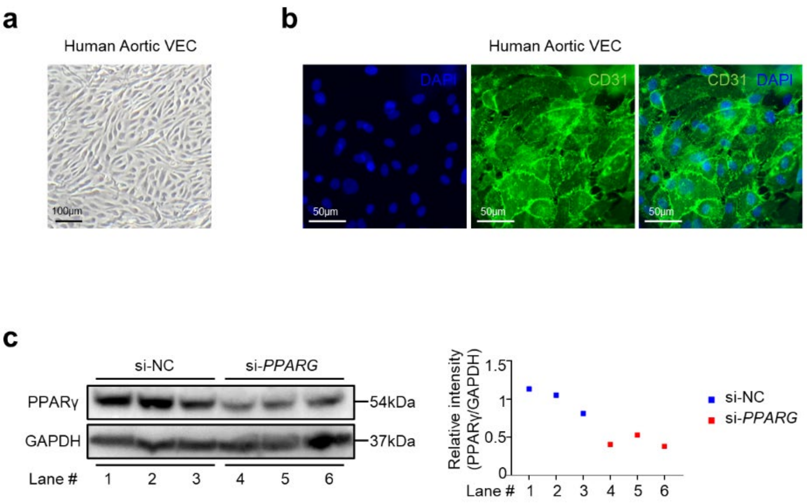

Supplementary Fig. 15. ***PPARG*** **gene silencing mediated by siRNA in human aortic VECs.**

a. Phase contrast microscopy image of cultured human aortic VECs at passage 3. Scale bar: 100 μm. **b.** Immunocytochemistry of CD31 (green) in human aortic VECs. DAPI (blue) was used for nuclei staining. Scale bar: 50 μm. **c.** *PPARG* gene knockdown by siRNA was validated by western blot (n = 3). The graph indicates the relative intensity of PPARγ to GAPDH. si-NC: negative control siRNA, si-*PPARG*: siRNA targeting *PPARG*. Image data are representative of at least three independent experiments.

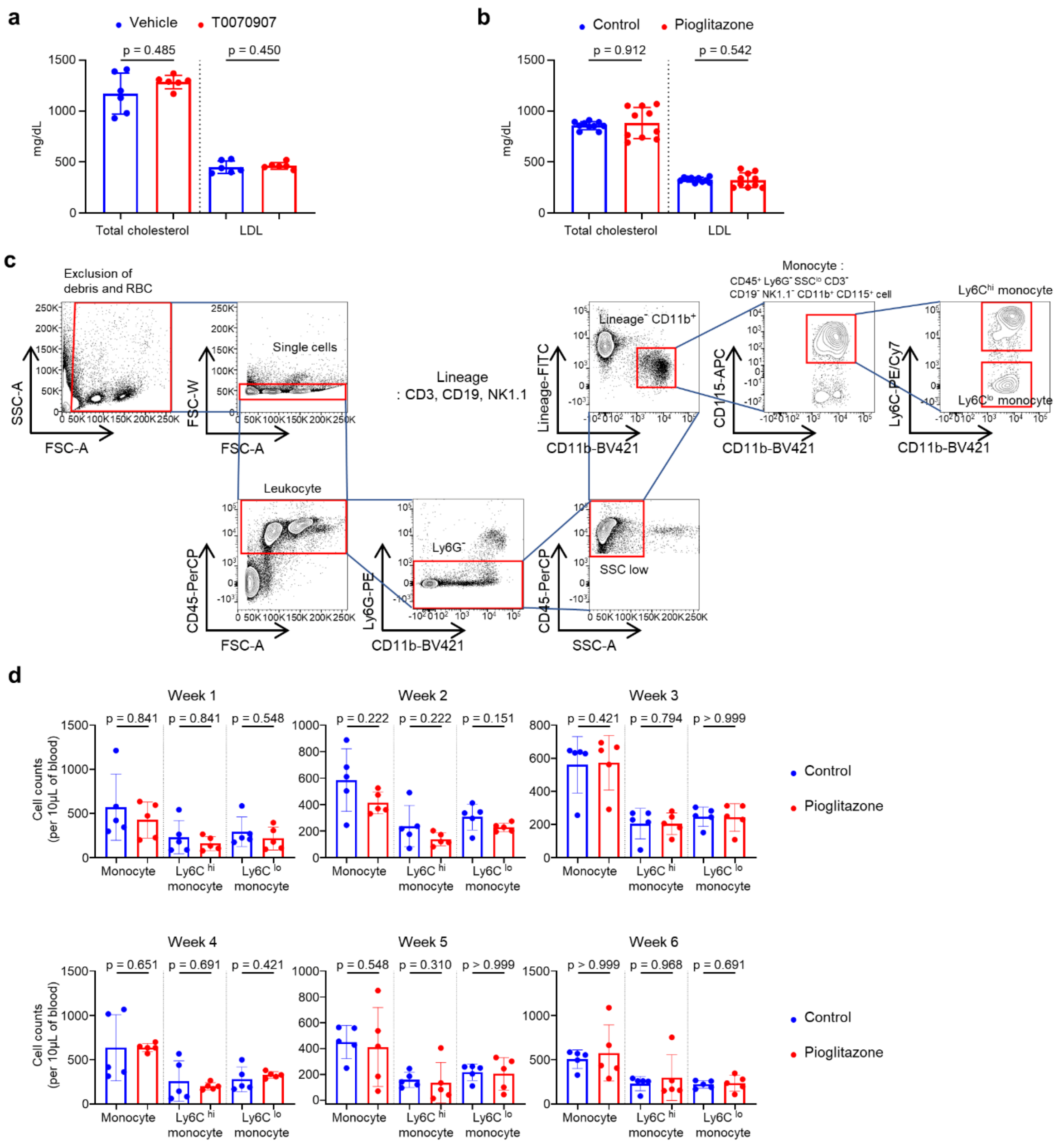

Supplementary Fig. 16. Effects of PPARG modulation in blood lipid profiles and the number of circulating monocytes.

a. Plasma total cholesterol and LDL levels of mice with T0070907 or vehicle administration (n = 6). **b.** Plasma total cholesterol and LDL levels of mice fed with pioglitazone-containing WD (pioglitazone) or normal WD (control) (n = 10). **c, d.** Flow cytometry analyses of circulating blood monocytes, for 6 weeks. PCSK9-AAV-injected C57BL/6J mice were fed

with pioglitazone-containing WD or normal WD for 6 weeks, and the number of monocytes and subsets in 10 μL of blood was examined once a week (n = 5). The gating strategy of blood monocytes and subsets. **(c)** and cell counts of monocytes, Ly6C high monocytes, and Ly6C low monocytes in 10 μL of blood, from week 1 to 6 **(d).** This figure is extended data of Fig. 8. Two-sided Mann-Whitney test was used for group comparisons. Data are presented as mean ± SD.

Group	ID	Age	Sex	Height (cm)	Weight (kg)	Comorbidity		Serum lipid profiles (mg / dL)			
						Hypertension	Diabetes mellitus	Total cholesterol	Triglyceride	HDL	LDL
Non-calcified	#1	67	M	160	56.7	Yes	No	208	247	38	122
	#2	67	F	152	62.1	Yes	No	273	325	54	153
	#3	73	M	162	67.9	Yes	No	126	110	45	31
	#4	71	M	166	64.6	Yes	No	195	153	63	90
	#5	64	M	157	59.9	No	No	132	73	85	91
	#6	53	M	176	91.9	Yes	Yes	118	220	40	63
	#7	52	M	170	69.4	No	No	105	56	25	75
	Mean ± SD	63.86 ± 8.295	N/A	163.3 ± 8.098	67.5 ± 11.63	N/A	N/A	165.3 ± 61.69	169.1 ± 98.95	50 ± 19.6	89.29 ± 39.63
Calcified	#1	76	M	161	62.6	Yes	No	151	90	41	108
	#2	75	M	161	63.4	Yes	No	146	183	41	87
	#3	85	M	163.9	60.8	Yes	No	171	78	51	102
	#4	75	F	157.4	67.5	Yes	No	138	96	58	64
	#5	61	F	156.8	60.3	Yes	No	148	277	33	70
	Mean ± SD	74.4 ± 8.591	N/A	160 ± 2.924	62.92 ± 2.858	N/A	N/A	150.8 ± 12.28	144.8 ± 84.83	44.8 ± 9.757	86.2 ± 19.24
Group comparison (p-value)		0.043	N/A	0.508	0.639	N/A	N/A	0.755	0.876	0.845	> 0.999

Supplementary Table 1. Patient clinical information of human sample used in histological analysis.

All patients provided informed consent, including to the publication of information that identifies individuals. Two-sided Mann-Whitney test was used for group comparisons (age, height, weight, and parameters of serum lipid profiles). M: male. F: female.

		C57BL/6J	Apoe -/-	Ldlr -/-
Sequencing	Number of Reads	46,539,591	151,825,684	181,607,997
	Q30 Bases in Barcode	96.2%	97.5%	97.5%
	Q30 Bases in RNA Read	84.9%	89.0%	89.1%
	Q30 Bases in Sample Index	94.7%	96.3%	95.3%
	Q30 Bases in UMI	96.3%	97.6%	97.6%
Mapping	Reads Mapped to Genome	95.7%	96.8%	96.8%
	Reads Mapped Confidently to Genome	92.2%	92.2%	91.9%
	Reads Mapped Confidently to Intergenic Regions	2.6%	2.1%	2.0%
	Reads Mapped Confidently to Intronic Regions	13.8%	9.4%	9.1%
	Reads Mapped Confidently to Exonic Regions	75.8%	80.8%	80.8%
	Reads Mapped Confidently to Transcriptome	73.6%	78.6%	78.7%
	Reads Mapped Antisense to Gene	0.9%	0.7%	0.7%
Cells	Mean Reads per Cell	14,575	94,477	85,502
	Median UMI Counts per Cell	3,351	6,708	5,514
	Median Genes per Cell	1,380	2,114	1,760
	Total Genes Detected	16,933	16,822	16,705
	Estimated Number of Cells	3,193	1,607	2,124
	Number of Quality Control Pass Cells	3,109	1,477	1,988

Supplementary Table 2. Summary of scRNA sequencing parameters.
