# Supplementary material for: Single-cell transcriptomics reveal cellular diversity of aortic valve and the immunomodulation by PPARγ during hyperlipidemia
Source: Nat Commun. 2022 Sep 17;13:5461. doi: 10.1038/s41467-022-33202-2 (PMC9482653; doi:10.1038/s41467-022-33202-2)
Supplement: Supplementary file 3 — Description of Additional Supplementary Files [file 41467_2022_33202_MOESM3_ESM.docx]

**Description of Additional Supplementary Files**

**File name: Supplementary Data 1**

Description: List of genes and significant (p<0.05) pathways specific to cell clusters of total valve cells, leukocytes, VECs, and VICs.

**File name: Supplementary Data 2**

Description: List of genes used for scoring myofibroblast activation and calcification.

**File name: Supplementary Data 3**

Description: List of genes specific to PPARγ regulon high versus low valvular cells and VECs.

**File name: Supplementary Data 4**

Description: List of differentially expressed genes from RNA-seq analysis of human aortic VECs.

**File name: Supplementary Data 5**

Description: List of antibodies used in this study.

**File name: Supplementary Movie 1**

Description: 3D movie of whole-mount aortic valves that ex vivo cultured without DiI-lipoproteins.

DAPI (blue) was used to stain nuclei. Red: autofluorescence. This movie is extended data of Fig. 1e. The movie is representative of three independent experiments.

**File name: Supplementary Movie 2**

Description: 3D movie of whole-mount aortic valves that ex vivo cultured with DiI-VLDL.

DAPI (blue) was used to stain nuclei. Red: VLDL. This movie is extended data of Fig. 1e. The movie is representative of three independent experiments.

**File name: Supplementary Movie 3**

Description: 3D movie of whole-mount aortic valves that ex vivo cultured with DiI-LDL.

DAPI (blue) was used to stain nuclei. Red: LDL. This movie is extended data of Fig. 1e. The movie is representative of three independent experiments.

**File name: Supplementary Movie 4**

Description: 3D movie of aortic valves of C57BL/6J with whole-mount immunofluorescence staining of MHC-II and CD206. MHC-II (red) and CD206 (green) signals were surface rendered. DAPI (blue) was used to stain nuclei. This movie is extended data of Fig. 3e. The movie is representative of three independent experiments.

**File name: Supplementary Movie 5**

Description: 3D movie of aortic valves of *Apoe*^-/-^ with whole-mount immunofluorescence staining of MHC-II and CD206. MHC-II (red) and CD206 (green) signals were surface rendered. DAPI (blue) was used to stain nuclei. This movie is extended data of Fig. 3e. The movie is representative of three independent experiments.

**File name: Supplementary Movie 6**

Description: 3D movie of aortic valves of *Ldlr*^-/-^ with whole-mount immunofluorescence staining of MHC-II and CD206. MHC-II (red) and CD206 (green) signals were surface rendered. DAPI (blue) was used to stain nuclei. This movie is extended data of Fig. 3e. The movie is representative of three independent experiments.
