# Supplementary material for: Single-cell transcriptomics reveal cellular diversity of aortic valve and the immunomodulation by PPARγ during hyperlipidemia
Source: Nat Commun. 2022 Sep 17;13:5461. doi: 10.1038/s41467-022-33202-2 (PMC9482653; doi:10.1038/s41467-022-33202-2)
Supplement: Supplementary file 15 — Reporting Summary [file 41467_2022_33202_MOESM15_ESM.pdf]

nature portfolio

Corresponding author(s): Hae-Ock Lee, Jae-Hoon Choi

Last updated by author(s): Aug 27, 2022

Reporting Summary

Nature Portfolio wishes to improve the reproducibility of the work that we publish. This form provides structure for consistency and transparency in reporting. For further information on Nature Portfolio policies, see our Editorial Policies and the Editorial Policy Checklist.

Statistics

For all statistical analyses, confirm that the following items are present in the figure legend, table legend, main text, or Methods section.

n/a	Confirmed	
☐	☒	The exact sample size (*n*) for each experimental group/condition, given as a discrete number and unit of measurement
☐	☒	A statement on whether measurements were taken from distinct samples or whether the same sample was measured repeatedly
☐	☒	The statistical test(s) used AND whether they are one- or two-sided<br>*Only common tests should be described solely by name; describe more complex techniques in the Methods section.*
☒	☐	A description of all covariates tested
☐	☒	A description of any assumptions or corrections, such as tests of normality and adjustment for multiple comparisons
☐	☒	A full description of the statistical parameters including central tendency (e.g. means) or other basic estimates (e.g. regression coefficient) AND variation (e.g. standard deviation) or associated estimates of uncertainty (e.g. confidence intervals)
☐	☒	For null hypothesis testing, the test statistic (e.g. *F*, *t*, *r*) with confidence intervals, effect sizes, degrees of freedom and *P* value noted<br>*Give P values as exact values whenever suitable.*
☒	☐	For Bayesian analysis, information on the choice of priors and Markov chain Monte Carlo settings
☒	☐	For hierarchical and complex designs, identification of the appropriate level for tests and full reporting of outcomes
☐	☒	Estimates of effect sizes (e.g. Cohen's *d*, Pearson's *r*), indicating how they were calculated

Our web collection on statistics for biologists contains articles on many of the points above.

Software and code

Policy information about availability of computer code

Data collection	NIS Elements (v4.30, Nikon), and ZEN (v8.1, Zeiss) were used to acquire microscopic image data. Evolution-Capt (v17.04a, Vilber Luormat) was used to acquire western blot image data. FACSDiva (v6.1.3, BD Biosciences) was used to acquire flow cytometry data.
Data analysis	NIS Elements (v4.30, Nikon), ImageJ (v1.53c, NIH), Imaris (v9.0.2, Bitplane), and Photoshop (v22.3.0, Adobe) were used to analyze microscopic image data. Evolution-Capt (v17.04a, Vilber Luormat) and ImageJ (v1.53c, NIH) were used to analyze western blot image data. FlowJo (v10.5.3, FlowJo, LLC) was used to analyze flow cytometry data. Cell Ranger toolkit (v3.0.2, 10x Genomics), R package Seurat (v3.1.1), Monocle (v2), ggplot2 (v3.3.3), stats (v3.6.3), ggpubr (v0.4.0), msigdbr (v7.1.1), fgsea (v1.12.0), and SCENIC (v1.1.2), RcisTarget (v1.6.0), AUCell (v1.8.0), and GENIE3 (v1.8.0) were used to analyze single-cell RNA sequencing data. STAR (v2.7.8a), RSEM (v1.3.1), and R package DESeq2 (v.1.26.0) were used to analyze the bulk RNA seq data. GraphPad Prism (v9.4.1, GraphPad) was used for statistical analysis.

For manuscripts utilizing custom algorithms or software that are central to the research but not yet described in published literature, software must be made available to editors and reviewers. We strongly encourage code deposition in a community repository (e.g. GitHub). See the Nature Portfolio guidelines for submitting code & software for further information.

Data

Policy information about availability of data

All manuscripts must include a data availability statement. This statement should provide the following information, where applicable:

- Accession codes, unique identifiers, or web links for publicly available datasets
- A description of any restrictions on data availability
- For clinical datasets or third party data, please ensure that the statement adheres to our policy

All data associated with this study are present in the main text or the supplementary materials. The RNA-seq data generated in this study have been deposited in

the GEO database under accession code "GSE180278 [https://www.ncbi.nlm.nih.gov/geo/query/acc.cgi?acc=GSE180278]", "GSE205587 [https://www.ncbi.nlm.nih.gov/geo/query/acc.cgi?acc=GSE205587]", and "GSE206927 [https://www.ncbi.nlm.nih.gov/geo/query/acc.cgi?acc=GSE206927]". The publicly available RNA-seq data used in this study are available in the BioProject database under accession code "PRJNA562645 [https://www.ncbi.nlm.nih.gov/bioproject/?term=PRJNA562645]", and in the ArrayExpress database under accession code "E-MTAB-7149 [https://www.ebi.ac.uk/arrayexpress/experiments/E-MTAB-7149/]", and "E-MTAB-8077 [https://www.ebi.ac.uk/arrayexpress/experiments/E-MTAB-8077/"]. "GRCh38 [https://ftp.ebi.ac.uk/pub/databases/gencode/Gencode_human/release_38/GRCh38.primary_assembly.genome.fa.gz]" and "GRCm38 [https://ftp.ebi.ac.uk/pub/databases/gencode/Gencode_mouse/release_M25/GRCm38.primary_assembly.genome.fa.gz]" were used for the reference genome. All other relevant data supporting the key findings of this study are available within the article and its Supplementary Information files or from the corresponding author upon reasonable request. Source data are provided with this paper.

Field-specific reporting

Please select the one below that is the best fit for your research. If you are not sure, read the appropriate sections before making your selection.

[x] Life sciences [ ] Behavioural & social sciences [ ] Ecological, evolutionary & environmental sciences

For a reference copy of the document with all sections, see nature.com/documents/nr-reporting-summary-flat.pdf

Life sciences study design

All studies must disclose on these points even when the disclosure is negative.

Sample size	The sample size was determined based on previous experiments of our group (https://doi.org/10.1161/CIRCRESAHA.118.312804, https://doi.org/10.1161/CIRCULATIONAHA.120.046907), and previous studies reported by other groups working on valvular disease and cardiovascular disease (https://doi.org/10.1161/ATVBAHA.112.300794, https://doi.org/10.1016/j.jacc.2005.09.049). We used the sample size of the in vivo and in vitro experiments as n≥3 individual samples for the proper statistical analysis to represent the sufficient reproducibility in vivo and in vitro experiments. One exception is the experiment using murine valvular interstitial cell (VIC) culture, which was n=2, but 10 mice pooled for each individual sample. We did not calculate sample-size calculations to predetermine the sample size.<br>The number of mice used in single-cell RNA sequencing was determined as for the visualization more than 5,000 cells in total. Based on this, the number of pooled mice in each experimental group is as follows: C57BL/6J (n=30), Ldlr-/- (n=24), and Apoe-/- group (n=19).
Data exclusions	Fig. 2: We failed to collect blood from one mouse of the PCSK9-AAV-infected group, therefore cannot produce lipid profile data in Fig. 2f, g.<br>Fig. 5k and Fig. 8a: In cell adhesion assay, samples that failed to appropriate whole-mounting on the slide were too hardly damaged to acquire data, and therefore excluded from the analysis.<br>For the quality control to our scRNA-seq analysis, we applied these criteria: (1) mitochondrial genes < 10%. (2) gene counts range from 200 to 5,000.
Replication	All replication of experiments was successful, and the validation of cell clusters using immunostaining or RNA in situ hybridization was faithfully confirmed the result of our scRNA-seq analysis. All data (except the data of Fig. 1g, which is n = 2) represent at least three independent experiments unless otherwise stated. The number of replication of the experiment is provided in the respective figure legend.
Randomization	In all experiments used in this study, the samples were divided randomly into each different group.
Blinding	Blinding was not applicable because of the evident morphological differences between the test group and the control group, and in this regard, blinding would not change the results in the data we collected.

Reporting for specific materials, systems and methods

We require information from authors about some types of materials, experimental systems and methods used in many studies. Here, indicate whether each material, system or method listed is relevant to your study. If you are not sure if a list item applies to your research, read the appropriate section before selecting a response.

Materials & experimental systems

n/a	Involved in the study	
[ ]	[x]	Antibodies
[x]	[ ]	Eukaryotic cell lines
[x]	[ ]	Palaeontology and archaeology
[ ]	[x]	Animals and other organisms
[ ]	[x]	Human research participants
[x]	[ ]	Clinical data
[x]	[ ]	Dual use research of concern

Methods

n/a	Involved in the study	
[x]	[ ]	ChIP-seq
[ ]	[x]	Flow cytometry
[x]	[ ]	MRI-based neuroimaging

Antibodies

Antibodies used	Detailed information is described in the Materials and Methods section and Supplementary Information.<br>Alexa Fluor 488 anti-mouse CD206 (Clone: C068C2, BioLegend, # 141709, Lot: B174661)<br>PE anti-mouse CD64 (Clone: X54-5/7.1, BioLegend, # 139304, Lot: B191540)

PerCP anti-mouse CD45 (Clone: 30-F11, BioLegend, # 103130, Lot: B236192)
PE/Cy7 anti-mouse/human CD11b (Clone: M1/70, BioLegend, # 101215, Lot: B176837)
Alexa Fluor 647 anti-mouse CD11c (Clone: N418, BioLegend, # 117314, Lot: B155267)
APC/Cy7 anti-mouse MHC class II (I-A/I-E), (Clone: M5/114.15.2, BioLegend, # 107628, Lot: B204231)
BV421 anti-mouse CD3 (Clone: 17A2, BioLegend, # 100228, Lot: B206350)
BV421 anti-mouse CD31 (Clone: MEC13.3, BD Biosciences, # 562939, Lot: 7306835)
FITC anti-mouse CD45 (Clone: 30-F11, BioLegend, # 103107, Lot: B293187)
PE/Cy7 anti-mouse F4/80 (Clone: BM8, BioLegend, # 123114, Lot: B200992)
APC/Cy7 anti-mouse/human CD11b (Clone: M1/70, BioLegend, # 101226, Lot: B222745)
PE anti-mouse CD140a (PDGFR-α) (Clone: APA5, BioLegend, # 135905, Lot: B244566)
APC anti-mouse CD140b (PDGFR-β) (Clone: APB5, BioLegend, # 136007, Lot: B256343)
APC/Cy7 anti-mouse PDPN (Podoplanin) (Clone: 8.1.1, BioLegend, # 127417, Lot: B253982)
PE/Cy7 anti-mouse Ly6C (Clone: HK1.4, BioLegend, # 128018, Lot: B175615)
PE anti-mouse Ly6G (Clone: 1A8, BioLegend, # 127607, Lot: B268001)
APC anti-mouse CD115 (Clone: AFS98, BioLegend, # 135509, Lot: B211308)
BV421 anti-mouse/human CD11b (Clone: M1/70, BioLegend, # 101251, Lot: B190213)
FITC anti-mouse CD3 (Clone: 17A2, BioLegend, # 100204, Lot: B172039)
FITC anti-mouse CD19 (Clone: 6D5, BioLegend, # 115505, Lot: B256057)
FITC anti-mouse NK-1.1 (Clone: PK136, BioLegend, # 108705, Lot: B252424)
PE Rat IgG2a, κ Isotype Ctrl Antibody (Clone: RTK2758, BioLegend, # 400508)
APC Rat IgG2a, κ Isotype Ctrl Antibody (Clone: RTK2758, BioLegend, # 400512)
APC/Cy7 Rat IgG2a, κ Isotype Ctrl Antibody (Clone: RTK2758, BioLegend, # 400523, Lot: B250268)
TruStain FcX™ (anti-mouse CD16/32) Antibody (Clone: 93, BioLegend, # 101320, Lot: B230968)
Anti-mouse/human PROX1 (Polyclonal, R&D Systems, # AF2727)
Anti-mouse CD36 (Polyclonal, R&D Systems, # AF2519, Lot: VYQ0220021)
Anti-mouse SR-B1 (Polyclonal, Novus Biologicals, # NB400-104, Lot: U)
Alexa Fluor 488 anti-goat IgG(H+L) (Polyclonal, Invitrogen, # A-11055, Lot: 1771339)
Cy3 anti-rabbit IgG(H+L) (Polyclonal, Jackson ImmunoResearch, # 711-165-152, Lot: 133645)
Anti-mouse CD68 (Clone: FA-11, Bio-Rad, # MCA1957)
Anti-mouse Vimentin (EPR3776, Abcam, # ab92547)
Cy3 anti-rat IgG(H+L) (Polyclonal, Jackson ImmunoResearch, # 712-165-153, Lot: 145787)
Alexa Fluor 647 anti-rabbit IgG(H+L) (Polyclonal, Invitrogen, # A-31573, Lot: 1964354)
HRP anti-rat IgG(H+L) (Polyclonal, Jackson ImmunoResearch, # 712-035-153, Lot: 136714)
Biotin anti-mouse MHC class II (I-A/I-E) (Clone: M5/114.15.2, BioLegend, # 107603, Lot: B189444)
Anti-mouse CD206 (Polyclonal, Abcam, # ab64693, Lot: GR3234777-1)
Anti-mouse/human PPARγ (Clone: C26H12, Cell Signaling Technology, # 2435, Lot: 6)
Anti-mouse Endomucin (EMCN) (Clone: V.7C7.1, Abcam, # ab106100, Lot: GR3270374-5)
Alexa Fluor 488 anti-rat IgG(H+L) (Polyclonal, Invitrogen, # A-21208, Lot: 2092264)
Anti-human CD31 (Clone: WM59, BioLegend, # 303102, Lot: B280539)
Alexa Fluor 488 anti-mouse IgG(H+L) (Polyclonal, Jackson ImmunoResearch, # 715-545-151, Lot: 148532)
Anti-human PPARγ (Clone: E-8, Santa Cruz Biotechnology, # sc-7273, Lot: K0520)
Anti-human GAPDH (Clone: 14C10, Cell Signaling Technology, # 2118, Lot: 14)
HRP anti-mouse IgG(H+L) (Polyclonal, BioLegend, # 405306, Lot: B329856)
HRP anti-rabbit IgG(H+L) (Polyclonal, Jackson ImmunoResearch, # 711-035-152, Lot: 139766)

Validation

All antibodies used in this study are commercially available and validated by the manufacturer of each antibody.
Alexa Fluor 488 anti-mouse CD206 (Clone: C068C2, BioLegend, # 141709, Lot: B174661): validated by manufacturer for species: mouse; application: flow cytometry, immunohistochemistry.
PE anti-mouse CD64 (Clone: X54-5/7.1, BioLegend, # 139304, Lot: B191540): validated by manufacturer for species: mouse; application: flow cytometry.
PerCP anti-mouse CD45 (Clone: 30-F11, BioLegend, # 103130, Lot: B236192): validated by manufacturer for species: mouse; application: flow cytometry.
PE/Cy7 anti-mouse/human CD11b (Clone: M1/70, BioLegend, # 101215, Lot: B176837): validated by manufacturer for species: mouse, human, cynomolgus, rhesus; application: flow cytometry.
Alexa Fluor 647 anti-mouse CD11c (Clone: N418, BioLegend, # 117314, Lot: B155267): validated by manufacturer for species: mouse; application: flow cytometry, immunohistochemistry, immunocytochemistry.
APC/Cy7 anti-mouse MHC class II (I-A/I-E), (Clone: M5/114.15.2, BioLegend, # 107628, Lot: B204231): validated by manufacturer for species: mouse; application: flow cytometry.
BV421 anti-mouse CD3 (Clone: 17A2, BioLegend, # 100228, Lot: B206350): validated by manufacturer for species: mouse; application: flow cytometry, immunocytochemistry.
BV421 anti-mouse CD31 (Clone: MEC13.3, BD Biosciences, # 562939, Lot: 7306835): validated by manufacturer for species: mouse; application: flow cytometry, immunofluorescence.
FITC anti-mouse CD45 (Clone: 30-F11, BioLegend, # 103107, Lot: B293187): validated by manufacturer for species: mouse; application: flow cytometry.
PE/Cy7 anti-mouse F4/80 (Clone: BM8, BioLegend, # 123114, Lot: B200992): validated by manufacturer for species: mouse; application: flow cytometry.
APC/Cy7 anti-mouse/human CD11b (Clone: M1/70, BioLegend, # 101226, Lot: B222745): validated by manufacturer for species: mouse, human, cynomolgus, rhesus; application: flow cytometry.
PE anti-mouse CD140a (PDGFR-α) (Clone: APA5, BioLegend, # 135905, Lot: B244566): validated by manufacturer for species: mouse; application: flow cytometry.

APC anti-mouse CD140b (PDGFR-β) (Clone: APB5, BioLegend, # 136007, Lot: B256343): validated by manufacturer for species: mouse; application: flow cytometry.
APC/Cy7 anti-mouse PDPN (Podoplanin) (Clone: 8.1.1, BioLegend, # 127417, Lot: B253982): validated by manufacturer for species: mouse; application: flow cytometry.
PE/Cy7 anti-mouse Ly6C (Clone: HK1.4, BioLegend, # 128018, Lot: B175615): validated by manufacturer for species: mouse; application: flow cytometry.
PE anti-mouse Ly6G (Clone: 1A8, BioLegend, # 127607, Lot: B268001): validated by manufacturer for species: mouse; application: flow cytometry.
APC anti-mouse CD115 (Clone: AFS98, BioLegend, # 135509, Lot: B211308): validated by manufacturer for species: mouse; application: flow cytometry.
BV421 anti-mouse/human CD11b (Clone: M1/70, BioLegend, # 101251, Lot: B190213): validated by manufacturer for species: mouse, human, cynomolgus, rhesus; application: flow cytometry.
FITC anti-mouse CD3 (Clone: 17A2, BioLegend, # 100204, Lot: B172039): validated by manufacturer for species: mouse; application: flow cytometry.
FITC anti-mouse CD19 (Clone: 6D5, BioLegend, # 115505, Lot: B256057): validated by manufacturer for species: mouse; application: flow cytometry.
FITC anti-mouse NK-1.1 (Clone: PK136, BioLegend, # 108705, Lot: B252424): validated by manufacturer for species: mouse; application: flow cytometry.
PE Rat IgG2a, κ Isotype Ctrl Antibody (Clone: RTK2758, BioLegend, # 400508): Isotype ctrl antibody, validated by manufacturer for application: flow cytometry.
APC Rat IgG2a, κ Isotype Ctrl Antibody (Clone: RTK2758, BioLegend, # 400512): Isotype ctrl antibody, validated by manufacturer for application: flow cytometry.
APC/Cy7 Rat IgG2a, κ Isotype Ctrl Antibody (Clone: RTK2758, BioLegend, # 400523, Lot: B250268): Isotype ctrl antibody, validated by manufacturer for application: flow cytometry.
TruStain FcX™ (anti-mouse CD16/32) Antibody (Clone: 93, BioLegend, # 101320, Lot: B230968): validated by manufacturer for species: mouse; application: flow cytometry.
Anti-mouse/human PROX1 (Polyclonal, R&D Systems, # AF2727): validated by manufacturer for species: human; application: western blot, immunocytochemistry. Usage for immunofluorescence in mouse sample was reported in previous studies (doi:10.1038/s41467-017-02796-3, doi:10.1172/JCI75395).
Anti-mouse CD36 (Polyclonal, R&D Systems, # AF2519, Lot: VYQ0220021): validated by manufacturer for species: mouse; application: western blot, flow cytometry, immunohistochemistry.
Anti-mouse SR-B1 (Polyclonal, Novus Biologicals, # NB400-104, Lot: U): validated by manufacturer for species: human, mouse, rat; application: western blot, flow cytometry, immunocytochemistry, immunofluorescence, immunohistochemistry.
Alexa Fluor 488 anti-goat IgG(H+L) (Polyclonal, Invitrogen, # A-11055, Lot: 1771339): validated by manufacturer for species: goat; application: immunohistochemistry, immunocytochemistry, flow cytometry.
Cy3 anti-rabbit IgG(H+L) (Polyclonal, Jackson ImmunoResearch, # 711-165-152, Lot: 133645): validated by manufacturer for species: rabbit; application: western blot, ELISA, immunohistochemistry, immunocytochemistry, flow cytometry.
Anti-mouse CD68 (Clone: FA-11, Bio-Rad, # MCA1957): validated by manufacturer for species: mouse; application: flow cytometry, immunofluorescence, immunohistochemistry, immunoprecipitation, western blot.
Anti-mouse Vimentin (EPR3776, Abcam, # ab92547): validated by manufacturer for species: mouse, rat, human; application: flow cytometry, immunocytochemistry, immunofluorescence, western blot, immunohistochemistry.
Cy3 anti-rat IgG(H+L) (Polyclonal, Jackson ImmunoResearch, # 712-165-153, Lot: 145787): validated by manufacturer for species: rat; application: western blot, ELISA, immunohistochemistry, immunocytochemistry, flow cytometry.
Alexa Fluor 647 anti-rabbit IgG(H+L) (Polyclonal, Invitrogen, # A-31573, Lot: 1964354): validated by manufacturer for species: rabbit; application: western blot, immunohistochemistry, immunocytochemistry, immunofluorescence.
HRP anti-rat IgG(H+L) (Polyclonal, Jackson ImmunoResearch, # 712-035-153, Lot: 136714): validated by manufacturer for species: rat; application: western blot, ELISA, immunohistochemistry, immunocytochemistry, flow cytometry.
Biotin anti-mouse MHC class II (I-A/I-E) (Clone: M5/114.15.2, BioLegend, # 107603, Lot: B189444): validated by manufacturer for species: mouse; application: flow cytometry. Usage for immunofluorescence in mouse sample was reported in the previous study (doi: 10.1038/s41541-017-0007-7).
Anti-mouse CD206 (Polyclonal, Abcam, # ab64693, Lot: GR3234777-1): validated by manufacturer for species: mouse, rat, human; application: immunohistochemistry, western blot, immunocytochemistry.
Anti-mouse/human PPARγ (Clone: C26H12, Cell Signaling Technology, # 2435, Lot: 6): validated by manufacturer for species: human, mouse; application: western blot, immunohistochemistry, immunofluorescence, chromatin immunoprecipitation.
Anti-mouse Endomucin (EMCN) (Clone: V.7C7.1, Abcam, # ab106100, Lot: GR3270374-5): validated by manufacturer for species: mouse, human; application: immunohistochemistry, immunoprecipitation, flow cytometry, western blot, immunocytochemistry, immunofluorescence.
Alexa Fluor 488 anti-rat IgG(H+L) (Polyclonal, Invitrogen, # A-21208, Lot: 2092264): validated by manufacturer for species: rat; application: immunohistochemistry, immunocytochemistry, immunofluorescence.
Anti-human CD31 (Clone: WM59, BioLegend, # 303102, Lot: B280539): validated by manufacturer for species: human, african green, baboon, cynomolgus, rhesus; application: flow cytometry, ELISA, immunocytochemistry.
Alexa Fluor 488 anti-mouse IgG(H+L) (Polyclonal, Jackson ImmunoResearch, # 715-545-151, Lot: 148532): validated by manufacturer for species: mouse; application: western blot, ELISA, immunohistochemistry, immunocytochemistry, flow cytometry.
Anti-human PPARγ (Clone: E-8, Santa Cruz Biotechnology, # sc-7273, Lot: K0520): validated by manufacturer for species: mouse, rat, human; application: western blot, immunoprecipitation, immunofluorescence, immunohistochemistry, ELISA.
Anti-human GAPDH (Clone: 14C10, Cell Signaling Technology, # 2118, Lot: 14): validated by manufacturer for species: human, mouse, rat, monkey, bovine, pig; application: western blot, immunohistochemistry, immunofluorescence, immunocytochemistry, flow cytometry.
HRP anti-mouse IgG(H+L) (Polyclonal, BioLegend, # 405306, Lot: B329856): validated by manufacturer for species: mouse; application: ELISA, western blot, immunohistochemistry.
HRP anti-rabbit IgG(H+L) (Polyclonal, Jackson ImmunoResearch, # 711-035-152, Lot: 139766): validated by manufacturer for species: rabbit; application: western blot, ELISA, immunohistochemistry, immunocytochemistry, flow cytometry.

Animals and other organisms

Policy information about studies involving animals; ARRIVE guidelines recommended for reporting animal research

Laboratory animals	Ldlr-/- mice, Apoe-/- mice, and Ccr2-/- mice were obtained from The Jackson Laboratory. Male C57BL/6J mice were purchased from SLC (Japan) or DBL (Korea). All mice were housed in the animal facility of Hanyang University under specific pathogen-free conditions in a 12-hour light/12-hour dark cycle with controlled temperature (20-24°C) and humidity (40-60%), and supplied with a normal chow diet and water ad libitum. All mice used in this study were male mice, and 8-10-week-old mice were gone on each experiment.
Wild animals	No wild animals were used in the study.
Field-collected samples	No field collected samples were used in the study.
Ethics oversight	All animal procedures were approved by the Institutional Animal Care and Use Committee (IACUC) of Hanyang University (certification numbers: HY-IACUC-22-0027 and HY-IACUC-22-0029) and conformed to its regulations.

Note that full information on the approval of the study protocol must also be provided in the manuscript.

Human research participants

Policy information about studies involving human research participants

Population characteristics	Tissue sections of human aortic valves from patients were used in this study. The state of samples: calcified, or non-calcified was determined by gross anatomy, then further validated by the histological observation. Information of covariates [age, sex, height, weight, comorbidity (hypertension and diabetes mellitus), and serum lipid profiles (total cholesterol, triglyceride, HDL, and LDL)] is presented in the Supplementary Table 1.<br>For human aortic VEC isolation, human aortic valves from two patients were provided by The Catholic University of Korea, Uijeongbu St. Mary's Hospital, and the information of patients is described in 'Human samples' section of Methods.
Recruitment	All donors are patients who went to surgery, recruited and managed by Yonsei Severance Hospital, Seoul National University Hospital, and Uijeongbu St. Mary's Hospital. There was no selection bias.
Ethics oversight	Experiments using human samples in this study were reviewed and approved by the Institutional Review Board (IRB) of Yonsei Severance Hospital, Seoul National University Hospital, and Uijeongbu St. Mary's Hospital. Human samples were provided by Yonsei Severance Hospital (Seoul, Korea, IRB No. 4-2018-0813), Seoul National University Hospital (Seoul, Korea, IRB No. 1104-122-360), and Uijeongbu St. Mary's Hospital (Uijeongbu, Korea, IRB No. UC19TIDE0142). All patients provided informed consent, including to the publication of information that identifies individuals.

Note that full information on the approval of the study protocol must also be provided in the manuscript.

Flow Cytometry

Plots

Confirm that:

- [x] The axis labels state the marker and fluorochrome used (e.g. CD4-FITC).
- [x] The axis scales are clearly visible. Include numbers along axes only for bottom left plot of group (a 'group' is an analysis of identical markers).
- [x] All plots are contour plots with outliers or pseudocolor plots.
- [x] A numerical value for number of cells or percentage (with statistics) is provided.

Methodology

Sample preparation	After euthanizing the mice with CO2, aortic valves were harvested and isolated into a single-cell suspension at 37 °C for 30 minutes by rotating incubation with Ca2+/Mg2+ DPBS containing collagenase II (1000 U/mL, Sigma-Aldrich, #C6885) and DNase I (90 U/mL, Roche, #10104159001).<br>For analysis of blood monocyte, peripheral blood was obtained from the retro-orbital sinus of live mice, and 10µL of blood was used for each sample. Red blood cells (RBCs) were lysed by RBC Lysis Buffer (eBioscience, #00-4333-57) according to the manufacturer's protocol.
Instrument	FACSAria III (for cell sorting, BD Biosciences), FACSCanto II (for analysis, BD Biosciences)
Software	FACSDiva (for data collection, v6.1.3, BD Biosciences), FlowJo (for data analysis, v10.5.3, FlowJo, LLC)
Cell population abundance	After debris exclusion, all live aortic valvular single-cells, without specific cell-type gating, were sorted. 64%, 61% and 48% of cells in total cells (C57BL/6J, Apoe-/- and Ldlr-/- mice respectively) were live single-cells. And more than 97% of efficiency showed via FACSDiva (BD Biosciences) program was monitored during cell sorting. Only dye for live/dead cell staining (PI) was used in cell sorting.
Gating strategy	FSC-A/SSC-A for debris removal, FSC-A/FSC-W or FSC-H for single-cell gating. PI or Zombie Aqua was used for live/dead cell staining and gating. We utilized fluorochrome-conjugated antibodies [Anti-target name (fluorochrome)] : CD45 (PerCP or

FITC) for gating leukocyte; CD64 (PE) & CD11b (PE-Cy7) or F4/80 (PE-Cy7) & CD11b (APC-Cy7) for gating macrophage; CD31 (BV421) & CD45 (PerCP or FITC) for gating valvular interstitial cell (VIC) and valvular endothelial cell (VEC); CD206 (Alexa Fluor 488) & MHC-II (APC-Cy7) for gating macrophage subsets; MHC-II (APC-Cy7) & CD11c (Alexa Fluor 647); CD3 (BV421) & MHC-II (APC-Cy7) for gating T cell; CD45 (PerCP), Ly6G (PE), CD11b (BV421), CD3 (FITC), CD19 (FITC), NK1.1 (FITC), CD115 (APC), Ly6C (PE-Cy7), and SSC-A for gating monocyte and subsets. The detailed gating strategy used in this study is described in Supplementary fig. 4, Supplementary fig. 6, and Supplementary fig. 15c.

☒ Tick this box to confirm that a figure exemplifying the gating strategy is provided in the Supplementary Information.
